# Capsid-specific removal of circulating antibodies to adeno-associated virus vectors

**DOI:** 10.1038/s41598-020-57893-z

**Published:** 2020-01-21

**Authors:** Berangere Bertin, Philippe Veron, Christian Leborgne, Jack-Yves Deschamps, Sophie Moullec, Yves Fromes, Fanny Collaud, Sylvie Boutin, Virginie Latournerie, Laetitia van Wittenberghe, Benoit Delache, Roger Le Grand, Nathalie Dereuddre-Bosquet, Olivier Benveniste, Philippe Moullier, Carole Masurier, Otto Merten, Federico Mingozzi

**Affiliations:** 1 Genethon and INSERM U951, 1 rue de l’internationale, 91000 Evry, France; 2 Atlantic Gene Therapies, Centre de Boisbonne, ONIRIS, La Chantrerie, BP 40706, 44307 Nantes, France; 3 Institute of Myology, Sorbonne University and INSERM U974, 105 boulevard de l’Hopital, 75013 Paris, France; 4 CEA, Université Paris-Sud 11, INSERM U1184, Immunology of Viral Infections and Autoimmune Diseases, IDMIT Department, IBFJ, Fontenay-aux-Roses, 92265 France; 5 Assistance Publique-Hôpitaux de Paris, 75013 Paris, France

**Keywords:** Molecular medicine, Translational research, Preclinical research

## Abstract

Neutralizing antibodies directed against adeno-associated virus (AAV) are commonly found in humans. In seropositive subjects, vector administration is not feasible as antibodies neutralize AAV vectors even at low titers. Consequently, a relatively large proportion of humans is excluded from enrollment in clinical trials and, similarly, vector redosing is not feasible because of development of high-titer antibodies following AAV vector administration. Plasmapheresis has been proposed as strategy to remove anti-AAV antibodies from the bloodstream. Although safe and relatively effective, the technology has some limitations mainly related to the nonspecific removal of all circulating IgG. Here we developed an AAV-specific plasmapheresis column which was shown to efficiently and selectively deplete anti-AAV antibodies without depleting the total immunoglobulin pool from plasma. We showed the nearly complete removal of anti-AAV antibodies from high titer purified human IgG pools and plasma samples, decreasing titers to levels that allow AAV vector administration in mice. These results provide proof-of-concept of a method for the AAV-specific depletion of neutralizing antibodies in the setting of *in vivo* gene transfer.

## Introduction

Adeno-associated virus (AAV) vectors are currently considered to be one of the most promising platforms for *in vivo* gene transfer^1^. The lack of pathogenicity of the wild-type virus, the ability to establish long-term transgene expression in post-mitotic cells, and the broad tissue and host range^2^ contributed to the wide adoption of AAV vectors in gene therapy^1^. Recombinant AAV of different serotypes have been successfully tested in human trials^3–9^, and a handful of AAV-based gene therapy products have been approved by the Food and Drug Administration and European Medicine Agency^10–12^. Despite these successes, some important limitations of the AAV technology remain^13^. In particular, pre-existing humoral immunity to the AAV capsid, which develops early in life upon exposure to the wild-type vector^14–16^, represents one of the most important obstacles to successful systemic vector administration in humans^17,18^. Results in mice^19^, non-human primates^20^ and humans^5^ indicate that even low levels of neutralizing antibodies can completely abrogate transduction even when relatively high doses of vector (exceeding 10^12^ vector genomes (vg)/kg) are infused through the bloodstream. Moreover, development of high-titer anti-AAV neutralizing antibodies following vector infusion would prevent re-dosing of AAV vectors, in case efficacy is lost over time^13,21^.

Pre-existing immunity to AAV resulting from exposure to the wild-type virus is highly prevalent in humans^22^, exceeding two thirds of the population for some AAV serotypes. So far, the approach to the problem of preexisting humoral immunity to AAV in clinical trials has been the exclusion of seropositive subjects from enrollment; however, this solution is far from being optimal, as it results in the exclusion of a significant proportion of otherwise potentially eligible patients. Several strategies have been proposed to address the issue of anti-AAV antibodies^17,18^. Among these, plasmapheresis is an attractive strategy as it represents a safe and clinically established technology broadly available^23^. Plasmapheresis is a process used to selectively remove blood components used to treat a variety of conditions including those caused by the acute overproduction of antibodies (e.g. autoimmunity, transplant rejection), in which removal of pathogenic immunoglobulins results in clinical benefit^23^.

In gene therapy with AAV vectors, plasmapheresis offers several potential advantages, including the fact that it is a relatively simple and risk-free procedure, and the fact that antibody depletion is only transient thus unlikely to result in prolonged immunosuppression. Few studies have explored the effect of plasmapheresis on anti-AAV titers. In humans undergoing plasmapheresis for the management of autoimmunity, repeated sessions of immune absorption reduced circulating anti-AAV antibody levels^24^. The reduction resulted in undetectable neutralizing antibody titers in subjects with low to moderate (1:20) pre-treatment titers, while an only partial decrease in anti-AAV titers was observed in subjects with high-level (e.g. 1:1000) pre-treatment antibodies. In the preclinical setting, non-specific plasma absorption of circulating IgG has also been explored as a strategy to remove anti-AAV antibodies^24–27^.

Despite its advantages, plasmapheresis is also characterized by potential drawbacks associated with the need for repeated cycles over time to completely eliminate detrimental antibodies and the lack of specificity of antibody removal, which inevitably can result in hypogammaglobulinemia, potentially exposing patients to an enhanced risk of infection^28^. In the setting of autoimmunity and transplant, purified human IgG (intravenous immunoglobulin, IVIg) is commonly supplied in combination with plasmapheresis to replenish the pool of circulating antibodies^28^. However, this would not be feasible in the setting of AAV gene transfer due to the fact that IVIg contains high-titer anti-AAV antibodies^29^.

Here, we first tested plasmapheresis as a strategy to lower anti-AAV antibodies in a non-human primate model of AAV gene transfer. We then developed an AAV-specific immune absorption column able to highly-efficiently deplete anti-AAV antibodies from a variety of plasma samples. Depletion of antibodies directed against the capsid did not affect total IgG levels and resulted in a dramatic decrease in neutralizing activity of plasma, even when the starting titer exceeded 1:1000. Matrix optimization and the use of empty AAV particles to capture antibodies further improved the efficacy of the approach as confirmed *in vivo* in a passive immunization model of gene transfer.

## Results

### Plasmapheresis allows for AAV vector readministration in non-human primates

**Figure 1:**
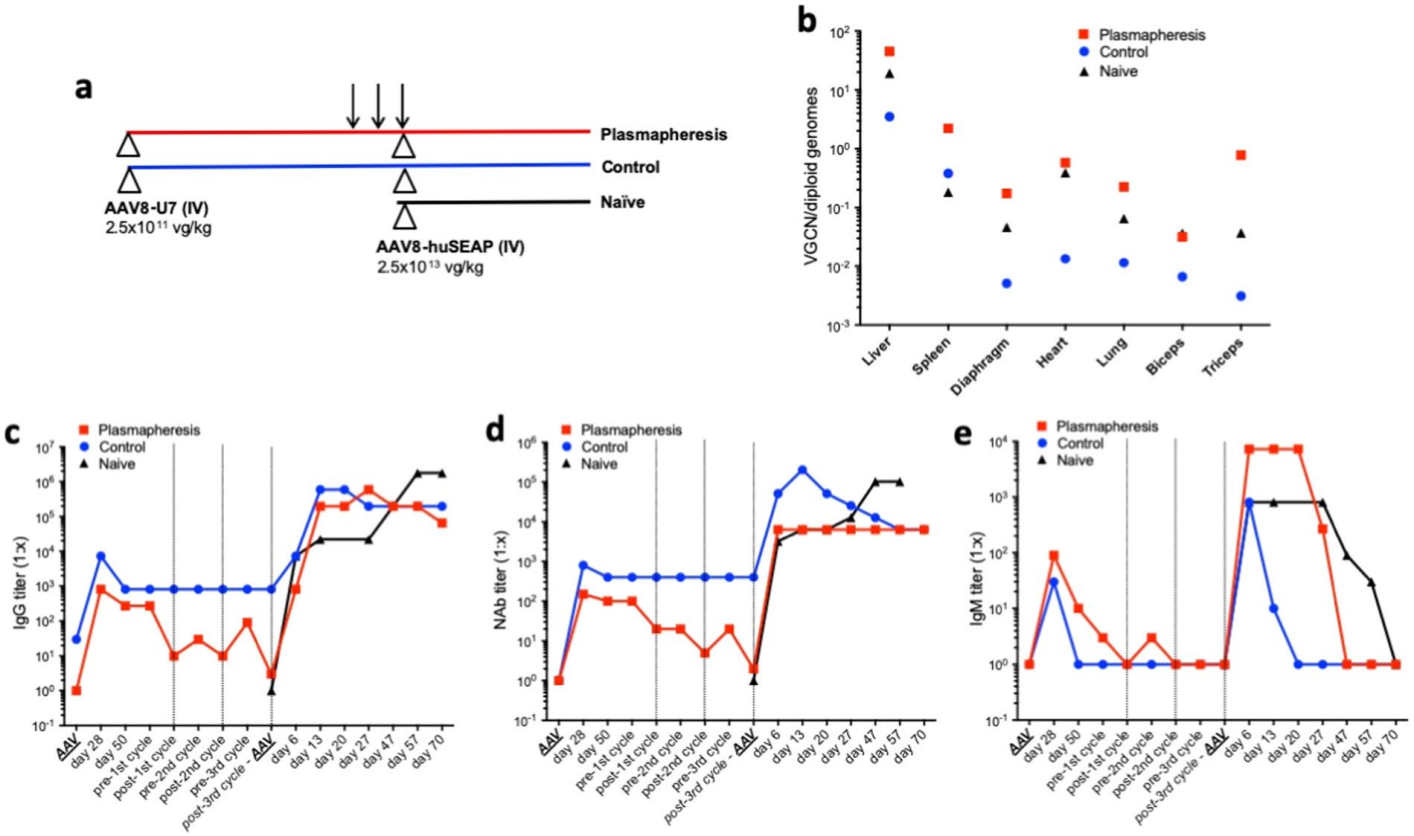
Plasmapheresis allows for AAV vector readministration in non-human primates. (**a**) Experimental design. Two animals received an AAV8-U7 vector intravenously at a dose of 2.5 × 10^11^vg/kg (Plasmapheresis and Control), 6 weeks later one animal (Plasmapheresis) was subjected to 3 cycles of plasmapheresis at a 2-day interval (arrows). Both animals and a third naïve control animal (Naïve) were then infused with an AAV8-huSEAP vector at a dose of 2.5 × 10^13^ vg/kg. Triangles indicate AAV vector administrations. (**b**) Vector genome copy number (VGCN) per diploid genome in various tissues collected at sacrifice. (**c–e**) Anti-AAV8 IgG (**c**), NAb (**d**), and IgM (**e**) levels over time. AAV in the x axis indicate the timing of AAV vector administration. Vertical dotted lines represent the timing of plasmapheresis cycles. Titers are reported as 1:x dilution.

As part of the evaluation of plasmapheresis as a technology to remove circulating anti-AAV neutralizing antibodies (NAbs), a pilot study was conducted in which two cynomolgus macaques received an AAV8 vector encoding for a U7 RNA specific for human dystrophin (AAV8-U7) at a dose of 2.5 × 10^11^ vg/kg administered intravenously (Fig. 1a). About 6 weeks later, when AAV NAb titers stabilized, one animal (labeled as Plasmapheresis) underwent 3 cycles of plasmapheresis 48 hours apart, which resulted in a 100-fold decrease in anti-AAV NAb titers, down to levels compatible with efficient AAV vector transduction^20^. Following the procedure, 2.5 × 10^13^ vg/kg of an AAV8 vector encoding for human secreted embryonic alkaline phosphatase (AAV8-huSEAP) were given intravenously. After each plasmapheresis cycle, the animal received an infusion of plasma from compatible animals with negative AAV NAbs titer to avoid excessive depletion of circulating immunoglobulins. The AAV8-huSEAP vector at the same dose was also administered to the immunized control animal which did not receive plasmapheresis (labeled as Control) and to a naïve animal with undetectable anti-AAV8 NAb titer (labeled as Naïve). All animals were followed for 70 days post AAV8-huSEAP administration and then sacrificed. Vector genome copy number (VGCN) specific for the AAV8-huSEAP vector was then evaluated in a variety of tissues (Fig. 1b). As previously published^25,27^, the Plasmapheresis animal displayed the highest VGCN in all tissues, closely followed by the Naïve animal. Conversely, as expected, the Control animal had the lowest VGCN in most tissues except for spleen (Fig. 1b), consistent with the expected biodistribution of AAV vectors in animals presenting anti-AAV antibodies at the time of vector administration^29,30^. The liver appeared to be the highest targeted organ, with VGCN in the Plasmapheresis animal ~1 log higher than the Control animal (Fig. 1b). Circulating levels of huSEAP in blood also confirmed the VGCN findings (Supplementary Fig. 1).

Anti-AAV antibody levels were also consistent with the VGCN results (Fig. 1c–e). Anti AAV-IgG and NAb titers decreased in the Plasmapheresis animal at each cycle of plasma absorption, down to levels close to preimmunization (Fig. 1c,d). Titers then increased again following the administration of the AAV8-huSEAP vector similar levels in all animals (Fig. 1c,d). As expected, IgM titers had an overlapping profile in all animals, characterized by a transient increase after each AAV vector administration (Fig. 1e).

These results confirm that plasmapheresis is a potentially useful technology to reduce pre-existing anti-AAV NAbs allowing for vector readministration, although it requires IgG supplementation to lower the risk of infection derived from the depletion of circulating antibodies.

### Grafting of capsid onto an NHS-Sepharose column results in efficient anti-AAV antibody capture

**Figure 2:**
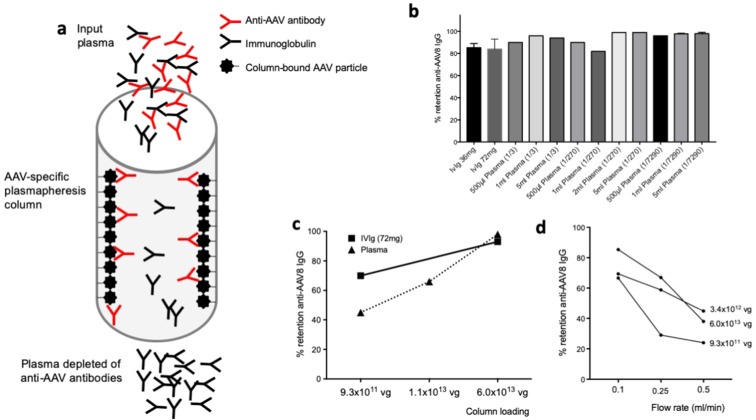
AAV-specific column for the removal of anti-capsid IgG from plasma. (**a**) Diagram of the column. AAV capsid particles are grafted to the column matrix and allow for binding and removal of IgG specific to the capsid without affecting total antibody levels. (**b**) Anti-AAV8 IgG retention rate of IVIg and plasma at various concentrations and volumes. A flow rate of 0.1 ml/minute was used in all experiments. For the IVIg retention experiment, n = 3 runs. For 1 ml and 5 ml plasma at 1/7290 dilution, n = 5 runs; shown is average, error bars represent the standard error of the mean. (**c**) Effect of AAV capsid loading on anti-AAV8 IgG retention. Various amounts of AAV8 capsid were used to bind to the NHS-Sepharose matrix and retention of AAV8 IgG measured after IVIg or seropositive plasma loading. (**d**) Effect of flow rate on anti-AAV8 IgG retention. AAV8 IgG retention tested with 3 columns containing various amounts of AAV8 capsid at 3 different flow rates.

Based on the promising results *in vivo* in non-human primates, we wanted to develop an improved technology to capture specifically anti-AAV antibodies. Toward this aim, we developed a series of chromatography columns based on commercially available matrices that we used to graft AAV capsid (Fig. 2a). Initially, we evaluated non-compressible chromatography matrices because they allow a relatively easy translation to larger scale, these included EPO-POROS (Epoxy activated chromatography support) in which the AAV capsid was grafted via EPO chemistry or crosslinking with formaldehyde. Both methods resulted in efficient capture of anti-AAV antibodies, although formaldehyde treatment resulted in lower retention of IgG (Supplementary Fig. 2). Based on published literature on plasmapheresis^31–33^, we then focused our attention on Sepharose, a compressible chromatography support. In a preliminary experiment, 3.4 × 10^12^ vg of AAV8 particles were crosslinked to 5ml N-Hydroxysuccinimidyl (NHS)-Sepharose. Leakage of vector genomes from the column was monitored using quantitative real-time PCR, showing only minimal leakage (~1 × 10^8^ vg) of viral particles after loading and four washes with PBS and sodium citrate followed by three empty runs (Supplementary Fig. 3). Next, three loads of intravenous immunoglobulin (IVIg, pooled human IgG containing anti-AAV IgG) or plasma were applied to the column at a flow rate of 0.5 ml/minute (0.1 CV/minute), and retention of capsid IgG was estimated using an anti-AAV8 IgG ELISA assay before loading, on flow through and after elution of the column (Fig. 2b). Retention >70% for IVIg and >80% for plasma of anti-AAV8 IgG was measured with different loads of IVIg as well as different volumes and dilutions of plasma (Fig. 2b), demonstrating efficient binding of anti-AAV8 antibodies to the AAV-Sepharose column.

Using the prototype column, we then evaluated the role of the density of the ligand on the efficiency of IgG retention (Fig. 2c). Three AAV8 densities were tested using a 5 ml volume of NHS-Sepharose, 9.3 × 10^11^, 1.1 × 10^13^, and 6.0 × 10^13^ vg/column. 72 mg of IVIg or 2 ml of human plasma positive for anti-AAV8 antibodies were loaded onto the columns and retention rate measured by ELISA. At the lowest AAV8 concentration, 9.3 × 10^11^ vg/column, retention of anti-AAV8 IgG was about 70% for IVIg. Possibly due to the presence of several proteins which may bind to AAV^34,35^, thus potentially interfering with the binding to the AAV capsid grafted to the column, only 45% of AAV8 IgG were retained when plasma was loaded to the column. Higher AAV8 concentrations resulted in better retention of antibodies, with >90% retention observed for both IVIg and plasma with the column containing 6.0 × 10^13^ vg/column AAV8 (Fig. 2c).

Next, we evaluated the effect of flow rate on the anti-AAV8 retention rate. Using three columns with 1 ml of resin grafted with 9.3 × 10^11^, 3.4 × 10^12^, or 6 × 10^12^ vg of AAV8, anti-AAV8 IgG retention was determined at flow rates of 0.1, 0.25, and 0.5 ml/min (0.02, 0.05, and 0.1 CV/min, Fig. 2d). As expected, higher flow rates resulted in lower retention of IgG in a setting a single passage of the IgG-containing fluid through the column. Repeated passages of IVIg through the same column or sequential passages through two columns enhanced removal of AAV8-specific IgG at any given flow rate (not shown).

These results support the feasibility of removing anti-AAV antibodies from plasma, they also establish some of the critical parameters affecting IgG retention efficiency.

### Empty AAV capsids retain IgG more efficiently than full capsids

**Figure 3:**
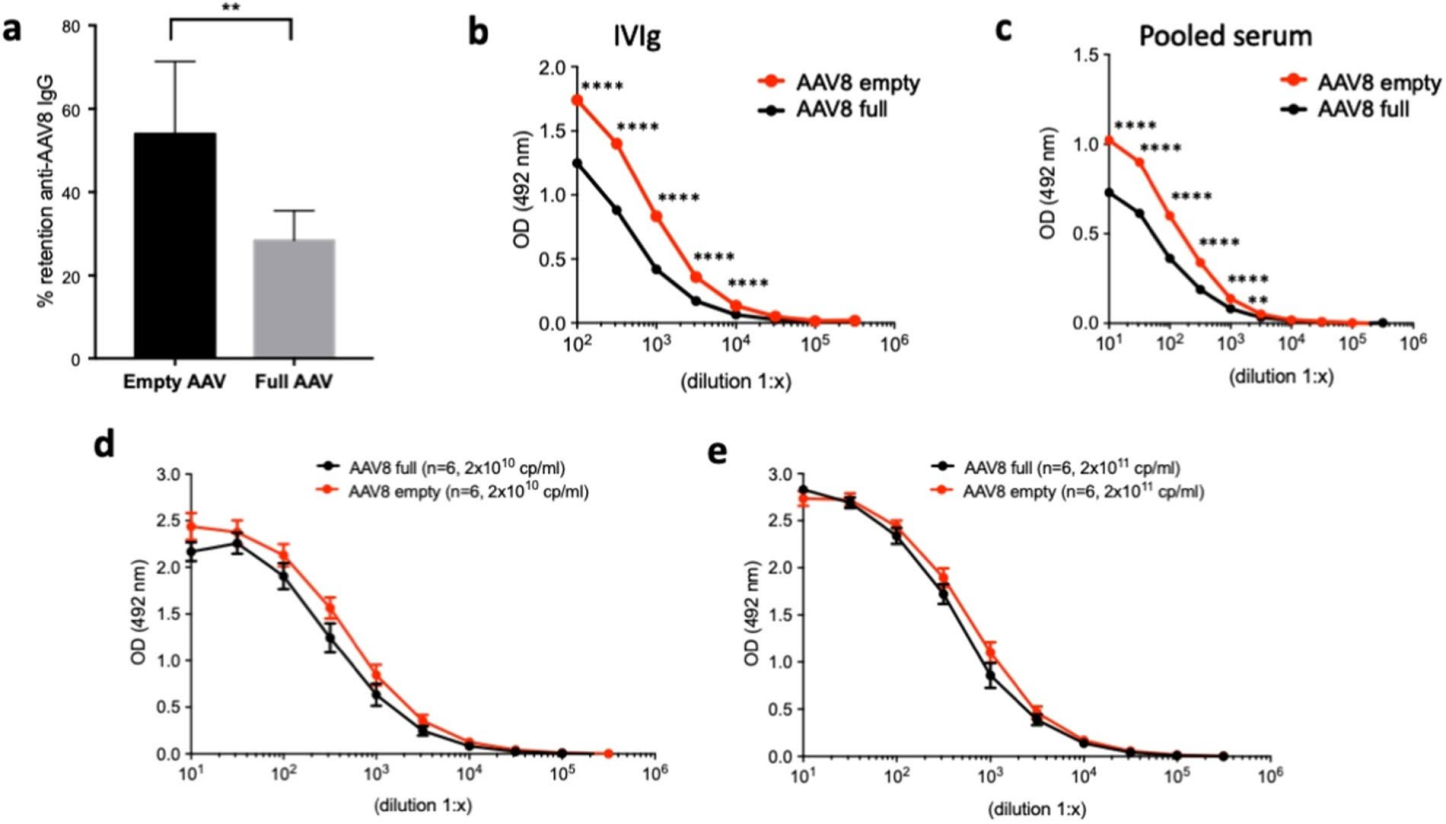
Binding of anti-AAV8 IgG to empty and full capsids. (**a**) AAV8 IgG retention following loading of 36 mg of IVIg loading onto NHS-Sepharose columns loaded with the same amount (2 × 10^12^ capsid particles, purified from the same AAV preparation) of empty vs. full capsids (empty AAV, n = 5 runs; full AAV, n = 8 runs). **p < 0.01 two-tailed unpaired t test. (**b,c**). ELISA assay to detect anti-AAV8 IgG in IVIg (**b**) or pooled human serum (**c**) at various dilutions. Plates were coated with 2 × 10^10^ capsid particles (cp)/ml of empty or full AAV8 capsid. (**d**,**e**). ELISA assay to detect anti-AAV8 IgG in IVIg using plates coated with either 2 × 10^10^ (**d**) or 2 × 10^11^ (**e**) cp/ml of empty or full AAV8 capsids deriving from n = 6 different preparations each. Error bars represent the standard error of the mean. Statistical analyses performed by RM two-way ANOVA, Sidak’s multiple comparisons test in panels (b,c) and ordinary two-way ANOVA, Sidak’s multiple comparisons test in panels (**d,e**). ****p < 0.0001; **p < 0.01.

In view of a possible use of an AAV-specific plasmapheresis column in humans, therefore to avoid the use of pharmacologically active AAV vectors in the plasmapheresis column, we tested whether empty capsids could be used to capture anti-AAV IgG. Multiple IgG retention experiments were performed with columns containing 2 × 10^12^ particles of either full (n = 8 runs) or empty (n = 5 runs) AAV capsids (Fig. 3a), showing a significantly more efficient retention of anti-AAV8 IgG with empty vs. full capsids (*p* < 0.01 t test) at a flow rate of 0.25 ml/min. To better investigate these findings, serial dilutions of both IVIg and human plasma were used in capture assays in which plates were coated with either empty or full AAV8 capsid (Fig. 3b,c). Detection of bound IgG with a secondary antibody confirmed the higher affinity of anti-AAV8 IgG for empty capsids. A series of similar tests with different preparations of empty and full capsids (n = 6 each) was then performed with IVIg using plates coated with 2 × 10^10^ or 2 × 10^11^ capsid particles/ml (Fig. 3d,e). In limiting conditions, i.e. in the presence of lower amounts of capsid coated onto the plates (2 × 10^10^ capsid particles/ml), empty capsids showed a higher, although not statistically significant, binding affinity for anti-AAV antibodies (Fig. 3d), which was not seen at higher capsid concentrations (Fig. 3e).

These results suggest that empty capsids bind anti-capsid antibodies more efficiently than full, potentially representing a better biological material for the development of AAV-specific plasmapheresis columns.

### AAV plasmapheresis columns specifically remove anti-AAV antibodies from plasma

**Figure 4:**
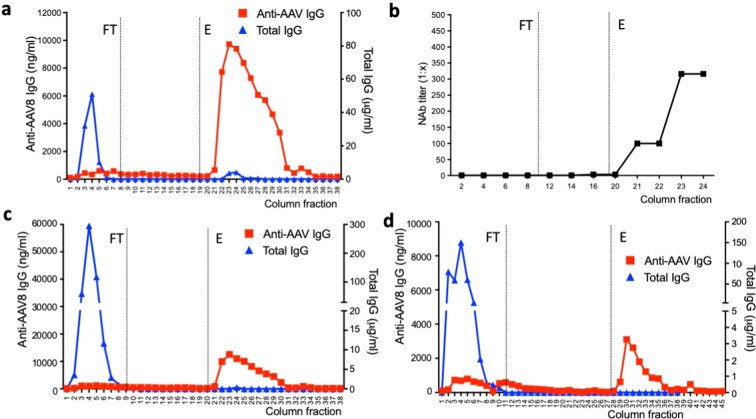
Antibody retention profile of AAV8-Sepharose columns. All columns contained full AAV capsids (2 × 10^12^ vg/1 ml of resin) (**a**) Anti-AAV8 IgG (red) and total IgG (blue) levels measured by ELISA in flow through (FT) and elution (E) fractions of an AAV8-Sepharose column after loading seropositive dog plasma (5 ml). (**b**) Anti-AAV8 NAb profile of the same dog sample across selected fractions from FT and E. NAb titers are reported as reciprocal dilution (1:x). Starting NAb titer before column treatment was >1:10,000. (**c**,**d**) Anti-AAV8 IgG (red) and total IgG (blue) levels measured in non-human primate (**c**) and human (**d**) plasma (loading volume 5 ml).

**Figure 5:**
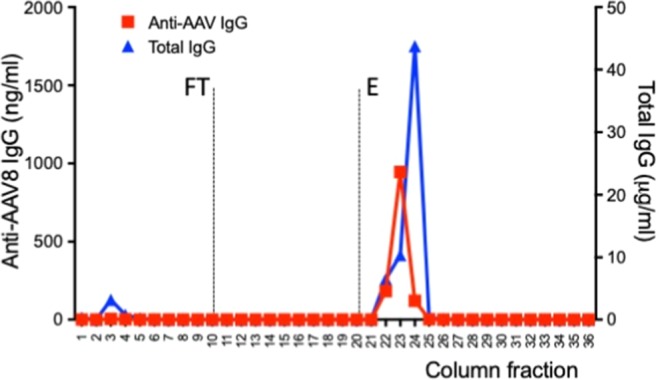
Antibody retention profile of a protein A column. Anti-AAV8 IgG (red) and total IgG (blue) levels measured by ELISA in flow through (FT) and elution (E) fractions of a protein A column after loading seropositive dog plasma (5 ml).

After optimization of the plasmapheresis column conditions, plasma samples of different origin were used to test efficiency and specificity of removal of capsid antibodies. Plasma was specifically used in these experiments as it was considered more representative of the clinical setting compared to that of purified IgG present in IVIg. First, plasma from a dog previously immunized with an AAV8 vector and displaying a baseline NAb titer of >1:10,000 was tested (Fig. 4a,b). The column was initially loaded with plasma and the flow through (FT) fractions of plasma were collected for analysis, showing lack of anti-AAV8 IgG and presence of total IgG. Conversely, when the column was eluted (E), only anti-AAV8 antibodies were detectable in the fractions collected (Fig. 4a). Accordingly, and in agreement with the published correlation between IgG and NAb titers^36^, only the elution fractions showed the presence of neutralizing antibodies against AAV8 (Fig. 4b). Consistent with a calculated ~30-fold dilution factor of the input seropositive plasma, the anti-AAV8 NAb titer in the elution fractions of the column peaked at 1:316 (Fig. 4b). Next, plasma from a non-human primate previously dosed with an AAV8 vector (Fig. 4c) and from a healthy donor pre-exposed to wild type AAV (Fig. 4d) were tested, showing similar specific retention of anti-capsid antibodies. NAb titers in the human plasma sample, which had a baseline NAb titer of 1:1,000, dropped to 1:3.16 in the FT fractions (after retention of the anti-AAV8 Ig), while NAb titers of 1:10 were found in the elution fractions. To compare these results with a column that was not antigen-specific, a protein A column was tested with a seropositive plasma sample (Fig. 5). As expected, virtually no IgG was released in the flow through (FT) at the time of passage of plasma through the column, while both total IgG and anti-AAV8 IgG were found in the fractions collected at the time of elution of the column (Fig. 5).

These results support the efficacy and specificity of AAV8 plasmapheresis columns in the removal of anti-capsid antibodies from plasma, even in the presence of high-titer antibodies derived from active immunization with AAV vectors.

### Column-mediated removal of anti-AAV8 IgG allows for efficient liver transduction **in vivo**

**Figure 6:**
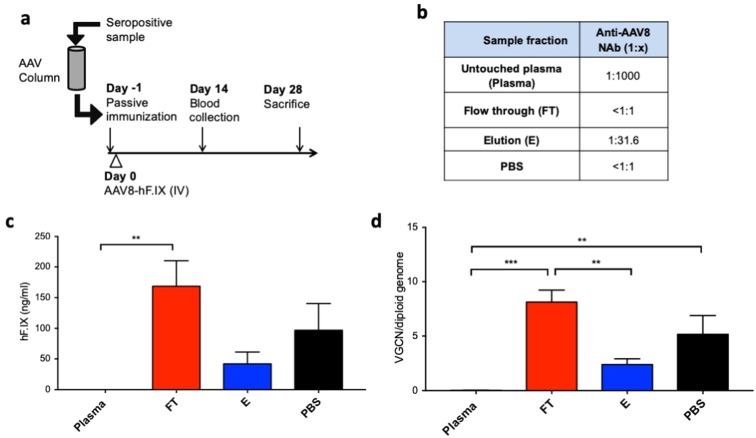
*In vivo* testing of removal of anti-capsid antibodies with the AAV8-Sepharose column. (**a**) Experimental design. A seropositive sample from a dog previously dosed with an AAV8 vector and displaying a titer of 1:1000 was subjected to column-based depletion of anti-AAV8 antibodies. Untouched plasma (Plasma), flow through (FT), elution (E) or PBS as control (PBS) were delivered to C57BL/6 mice (100 ul/mouse, n = 5/6 per treatment group) on day −1. On day 0 5 × 10^10^ vg/mouse of AAV8-hF.IX vector were delivered intravenously. Plasma levels of hF.IX were measured at day 14 and vector genome copy number (VGCN) were measured in liver at day 28. (**b**) Anti-AAV8 NAb profile of samples delivered to mice on day -1. (**c**) hF.IX plasma levels in mice at day 14 post vector administration. (**d**) VGCN per diploid genome in liver of mice at sacrifice. Results in (**c**,**d**) are shown as average, error bars represent the standard error of the mean. **p < 0.01; ***p < 0.001 one-way ANOVA.

To confirm our findings *in vivo*, we used a previously published passive immunization mouse model^37^. Seropositive plasma was passed through an AAV-Sepharose column and the different fractions of the column were subsequently infused intravenously in C57BL/6 mice. The day after C57BL/6 mice (n = 5/6 per group) received an AAV8 vector encoding for coagulation factor IX (AAV8-hF.IX) at a dose of 5 × 10^10^ vg/mouse. Animals were followed up for 4 weeks (Fig. 6a). Before column treatment, the plasma sample used for the passive immunization experiment presented a high NAb titer (Plasma, NAb titer 1:1000), which decreased to undetectable in the flow through (FT, NAb titer < 1:1) of the column (Fig. 6b). As observed in previous experiments, the anti-AAV8 NAbs were then found in the elution fraction of the column at a titer decreased due to the ~30-fold dilution of the sample (E, NAb titer 1:31.6, Fig. 6b).

Animals were passively immunized with Plasma, FT or E fractions, or PBS as control and after vector delivery hF.IX transgene levels in plasma were determined by ELISA. No transgene expression was observed in animals receiving untouched plasma, while animals receiving the FT fraction of the column had significantly higher transgene expression levels (Fig. 6c). Accordingly, vector genome copy number (VGCN) in livers collected at day 28 post vector infusion showed significantly higher levels of transduction of hepatocytes in FT-treated mice compared to Plasma or E treated animals (Fig. 6d).

These results confirm, in an *in vivo* model of passive immunization, that pretreatment of plasma with an AAV-specific antibody binding column results in rescue of liver transduction following AAV vector administration.

## Discussion

Pre-existing anti-AAV antibodies are one of the main obstacles to the efficient systemic delivery of AAV vectors. Antibodies originated from exposure to wild-type AAV, or deriving from administration of AAV vectors, can in fact neutralize AAV vectors, resulting in lack of efficacy^5^. Consequent to humoral immune responses to AAV, a significant proportion of seropositive participants is excluded from AAV vector-based trials, while vector redosing, in case needed, is virtually impossible without immunomodulation at the time of gene transfer^38,39^. Here, we initially tested the efficiency of plasmapheresis in the removal of anti-AAV antibodies in non-human primates. Like previously described^25^, we showed rescue of tissue transduction following systemic AAV vector, although IgG-containing plasma supplementation was necessary to maintain the levels of total circulating IgG sufficient to prevent infections. Next, to improve the safety and the efficacy of plasmapheresis to remove anti-capsid antibodies, we developed an AAV-specific plasmapheresis column for the selective removal of anti-AAV antibodies from the bloodstream. By optimizing column conditions, and defining the parameters critical for antibody absorption, we achieved nearly complete removal of vector-specific antibodies from pooled purified IgG (IVIg) preparations and from various plasma samples after a single depletion passage. We showed that removal of capsid antibodies was selective, as the total pool of circulating IgG was not depleted; furthermore, we showed that plasma samples passed through the column lost their ability to neutralize AAV vectors *in vivo* in a murine model of passive immunization.

Plasmapheresis is routinely used for the physical removal of antibodies from the circulation. The advantage of plasmapheresis over pharmacological approaches targeting B cells^40^ is the high efficiency in the removal of antibodies and the fact that the effect is only transient as it does not involve targeting antibody producing cells with immunosuppressive drugs. The technology is indicated in several autoimmune diseases and to prevent antibody-mediated graft rejection^23^. Few publications can be found in the literature on the use of plasmapheresis in the context of AAV gene transfer, including preclinical studies in non-human primates^25,26^ suggesting that the technology is effective in removing anti-AAV antibodies. To this aim, Monteilhet and colleagues^24^ showed that repeated cycles of plasma absorption in autoimmune disease patients results in the reduction of anti-AAV antibody titers, although this requires several subsequent sessions to efficiently eliminate both vascular and extravascular antibodies to AAV. Interestingly, in this study 5 cycles of plasmapheresis were not effective in completely eliminating high-titer neutralizing antibodies^24^, thus suggesting the need for more cycles of plasma absorption. While in theory it is possible to repeat immune absorption cycles until all antibodies are eliminated, this would be associated with prolonged periods of hypogammaglobulinemia and potential exposure to infections. The strategy presented here, based on the selective removal of anti-AAV antibodies, present a clear improvement over the conventional nonspecific technology as it would allow for repeated anti-AAV antibody depletion cycles with no effect on total IgG levels. In the context of conventional plasmapheresis, a decrease of IgG levels below a critical level would require supplementation with pooled human IgG, which by default contain high titer anti-AAV NAbs. One possible solution to the issue, which was also implemented here to supplement IgG in the non-human primate undergoing conventional plasmapheresis, is the supplement of IgG from AAV seronegative subjects, or else the development of AAV antibody-negative IgG pools. However, both approaches are unlikely to be feasible, as anti-AAV antibodies are highly prevalent in humans^22^.

One somewhat unexpected result obtained in the current study is the higher affinity of anti-AAV antibodies for empty capsids. The explanation of the finding not entirely unclear, although it is known that empty and full capsid are not identical, based on electron microscopy images of empty and full capsids^41^ and on the fact that it is possible to design purification methods able to discriminate between empty and full capsids^42^, suggesting both some underlying biological and structural differences driving the different binding to circulating antibodies. One possibility is that, due to the absence of the DNA genome, empty capsids could have a higher flexibility allowing a more intense antigen-antibody interaction than full AAV capsid particles. In this context, Carrasco and colleagues^43^ using the minute virus of mice (MVM, a parvovirus) were able to show specific protein-DNA interactions in the case of full capsids owing to an anisotropic increase in the mechanical capsid stiffness. Whether a similar model apply also to interactions of antibodies with the AAV capsid remains to be elucidated, along with binding affinity studies. Clearly, empty capsids are the most suitable material to be used to develop a plasmapheresis column, as they are inert due to the lack a genome.

While the data presented here are promising, additional work will be needed to include the technology presented here in a plasmapheresis device and to test the strategy in large animal models that are natural host for AAV, like non-human primates^44^, to mimic the context of AAV immunity found in humans and the actual plasmapheresis conditions used in the clinic. Critical parameters, like the optimal quantity of capsid antigen required to efficiently capture anti-AAV Ig, will also need to be defined. Furthermore, it would be useful to identify protocols in which the current technology is combined with an immunomodulatory regimen to increase the efficiency of the approach and thus achieve clearance of anti-AAV antibodies more rapidly than with plasmapheresis alone^45^. While the current proof-of-concept work demonstrated the feasibility of the approach in the context of one specific serotype, the development of column containing multiple AAV serotypes, therefore with different specificities, could potentially represent a universal device for antibodies directed to different AAVs. Additionally, as data on domains within the AAV capsid that are also epitopes of neutralizing antibodies become available, it may be possible to develop an AAV-free plasmapheresis column only based on specific peptides.

One open question about the technology described here is about the potential risk of triggering immunotoxicities associated with reactivation of memory cytotoxic T responses directed against transduced cells^18^. In the setting of AAV vector redosing, immunosuppression with corticosteroids^3,8^, other T cell-targeting drugs^46^, or the use of agents targeting both B and T cell responses to AAV^39^, used in combination with anti-AAV Ig depletion, may be required. Given the lack of correlation between humoral and cell-mediated immunity in the context of natural immunity to wild-type AAV^36,47,48^, the need for immunomodulation in the setting of eradication of naturally occurring anti-AAV antibodies is less understood.

In summary, here we presented a strategy based on AAV-specific plasmapheresis for the selective removal of circulating antibodies that can neutralize AAV vectors. The approach appeared to remove antibodies efficiently and specifically, without affecting total IgG levels. This work provides proof-of-concept of the feasibility of the approach and its potential using as an enabler of systemic gene transfer *in vivo* when anti-vector antibodies are present.

## Materials and Methods

### Specimens and animal experiments

All plasma samples of human origin were purchased from the French blood bank (Etablissement Français du Sang, EFS, Rungis, France). Intravenous immunoglobulin (IVIg) was purchased from LFB Biomedicaments (Courtaboeuf, France), the same lot of IVIg was used across all studies shown in this manuscript. All samples from pre-immunized animals came from previous studies approved by the local ethical committees.

Male C57BL/6 mice (males, 8–10 weeks of age) were obtained from Charles River (St. Germain Nuelles, France). Mouse studies were performed in accordance to the current European legislation on animal care and experimentation (2010/63/EU) and approved by the institutional ethical committee of the Centre d’Exploration et de Recherche Fonctionnelle Expérimentale (protocol number APAFIS 3055-20151019213299180).

Adult cynomolgus macaques (Macaca fascicularis) were imported from Noveprim Europe and housed at the facilities of the Centre de Boisbonne, Ecole Nationale Vétérinaire de Nantes (ONIRIS, Nantes). The plasmapheresis protocol was reviewed by the Ethical Animal Committee and registered by the French Research Ministry. Blood collection for the isolation of seronegative plasma was approved by the Ethical Committee number 44 of CEA (French Alternative Energies and Atomic Energy Commission), study number A13_017.

### AAV vectors

The AAV8 vector expressing human secreted embryonic alkaline phosphatase (AAV8-huSEAP) and the AAV8-U7 vector^49^ were produced in a baculovirus system^50^. The AAV8 vector encoding for human factor IX (AAV8-hF.IX) was previously described^37^, this vector and the preparations of full and empty vector particles were made as previously described^51^. All vectors were titrated using a quantitative real-time PCR assay. Empty AAV capsids were quantified using SDS-PAGE after Sypro Ruby staining (Molecular Probes, Thermo Fisher Scientific, Waltham, MA). Gel images were acquired on a gel reader G:box HR (Syngene, Frederick, MD) and the bands corresponding to the VP3 capsid protein quantified against a standard of known concentration using the software Image studio light (LI-COR Biosciences, Lincoln, NE).

### Preparation of the column matrices

Two different columns matrices, one based on the compressible NHS-Sepharose (HiTrap NHS activated, N-hydroxysuccinimide activated, GE HealthCare, St. Quentin en Yvelines, France) and one based on the incompressible EPO-POROS 50 resin (POROS EP450, 50 nm, Thermo Fisher Scientific, Waltham, MA), were initially evaluated in this study. For the NHS-Sepharose, the ligand density was 10 µmol ligand/ml of gel according the manufacturer directions; for the EPO-POROS support, the ligand density was determined empirically. Full or empty AAV particles formulated in PBS were grafted to the supports following the manufacturers’ recommendations. For EPO-POROS, 3.7 × 10^11^ particles of AAV8 capsid were added to 1 ml of resin. Coupling was performed at 4 °C for 60 hours. Residual active sites were inactivated by incubation with ethanolamine (1 M in a 0.5 M phosphate buffer, pH 9.0) for 2 hours at room temperature followed by washing with PBS. In order to reduce potential leaching of coupled AAV particles, crosslinking with formaldehyde (37% diluted 1/200 in PBS) was performed at 4 °C for 15 days. Then, residual formaldehyde was inactivated by incubation with Na-meta-bisulphite (3.75%, diluted 1/100 in PBS) for 2 hours at room temperature, followed by washing with PBS. For NHS-Sepharose, different amounts of AAV8 capsid ranging from 9.3 × 10^11^ to 6.0 × 10^13^ particles were coupled with 5 ml of HiTrap NHS support. After 1 hour of incubation at room temperature, residual active sites were inactivated by incubation with ethanolamine for 30 minutes at room temperature followed by washing with PBS. At the time of grafting of AAV particles to a column, four washes with PBS and sodium citrate at pH 4 were performed to eliminate unbound capsid (Supplementary Fig. 3).

### Column conditions for removal of anti-AAV antibodies from biological samples

All chromatography runs were performed on an ÄKTA explorer chromatography system (GE HealthCare, Uppsala, Sweden). UV (280 nm, 260 nm), pH, conductimetry (mS) and flow rate were monitored during each run. For the EPO-POROS columns, the tests were performed with either a 1.4-fold dilution of IVIg (to a concentration of IgG of 35.7 mg/ml) or undiluted plasma. A flow rate of 0.5 ml/min (0.5 column volume (CV)/min) was applied. For the NHS-Sepharose columns, various volumes of diluted IVIg (35.7 mg/ml) or undiluted plasma samples were used as indicated in the results section. Different flow rates ranging from 0.5 ml/min (0.1 CV/min) to 0.1 ml/min (0.02 CV/min) were initially tested and a rate of 0.1 ml/min (0.02 CV/min) was then selected. For the HiTrap Protein A HP (5 ml, GE HealthCare, Uppsala, Sweden) different volumes (1 ml, 2 ml, 3 ml and 5 ml) of either IVIg (35.7 mg/ml) or plasma were used. The buffer system used was PBS (running and washing buffer) for all columns. The volume of wash buffer (PBS) used was 12 ml for all columns. Similarly, elution was performed identically for all columns using 12 ml of 50 mM citrate buffer (pH 3.0). All experimental conditions were tested at least 3 times.

### Human factor IX transgene expression

Human F.IX expression in mice sera was determined by ELISA according to a previously published protocol^37^.

### Antibody titer determination

The anti-AAV8 IgG ELISA was previously described^29^. Briefly, plates were coated with AAV8 capsid (1 × 10^10^ capsid particles/ml, 50 µl per well, unless indicated otherwise), blocked with PBS with 6% non-fat dry milk, and serum or plasma samples added to the wells in duplicate. The levels of total IgG in plasma were determined by ELISA as previously described^22^. The *in vitro* AAV vector neutralizing antibody assay was performed as previously described^52^. The citrate buffer used to elute anti-AAV IgG bound to the column did not appear to influence the NAb assay performance.

### Vector genome copy number determination

Tissues biopsies were taken from various organs. One biopsy per tissue was weighed and homogenized in 500–700 µl lysis buffer and genomic DNA was isolated according to the manufacturer’s protocol (Puregene Blood Core kit, Qiagen, Hilden, Germany) and quantified using a Nanodrop ND-8000 spectrophotometer (Thermo Fisher Scientific, Courtaboeuf, France), 100 ng of genomic DNA were used in each reaction in triplicate. Vector genome copy numbers per cell were quantified by real-time quantitative PCR as previously described^39^. Primers and probes used were as follows: huSEAP forward primer, 5′-CCTAGGTGGAGGCCGAAAG-3′; huSEAP reverse primer: 5′-TGGTCCCACCTTGGCTGTA-3′; huSEAP probe: 5′-AACCCCAGACCCTGAGTACCCAGATGA-3′. The primers and probe to detect the AAV8-hF.IX vector genomes were previously described^29^.

### Statistical analysis

Statistical analyses were performed using the Prism software version 7.0a (GraphPad Software, Inc., San Diego, CA). Statistical methods used to compare datasets are specified in the text. *p* values below 0.05 were considered significant.

Data generated or analysed during this study are included in this published article (and its Supplementary Information files) or are available from the corresponding author on reasonable request.

## Supplementary information

Supplementary information.
