# Supplementary material for: Capsid-specific removal of circulating antibodies to adeno-associated virus vectors
Source: Sci Rep. 2020 Jan 21;10:864. doi: 10.1038/s41598-020-57893-z (PMC6972890; doi:10.1038/s41598-020-57893-z)

**Supplementary Figure 1**

Plasmapheresis allows for AAV vector readministration in non-human primates. huSEAP levels in serum post vector administration. Results are shown as the average of triplicate testing, error bars represent the standard error of the mean.

**Supplementary Figure 2**

Comparison of IgG retention rate of columns containing native AAV vs. formaldehyde crosslinked AAV capsid. Shown are recovery rates for different plasma samples loaded in parallel onto the two columns. Loading 5ml per sample. ***p*<0.01, Wilconox matched pairs test.

**Supplementary Figure 3**

Release of AAV particles from the AAV-Sepharose colum at capsid grafting, washing, and during test runs. Table, vector genome (vg)/ml, determined by real-time quantitative PCR and total vg determination based on the volume the fractions. Chart, graphical representation of the loading and total vg loss per fraction. Run 0, wash steps following loading. Citrate, wash with sodium citrate pH 4.

**Supplementary Figures**

**Supplementary Figure 1**

**
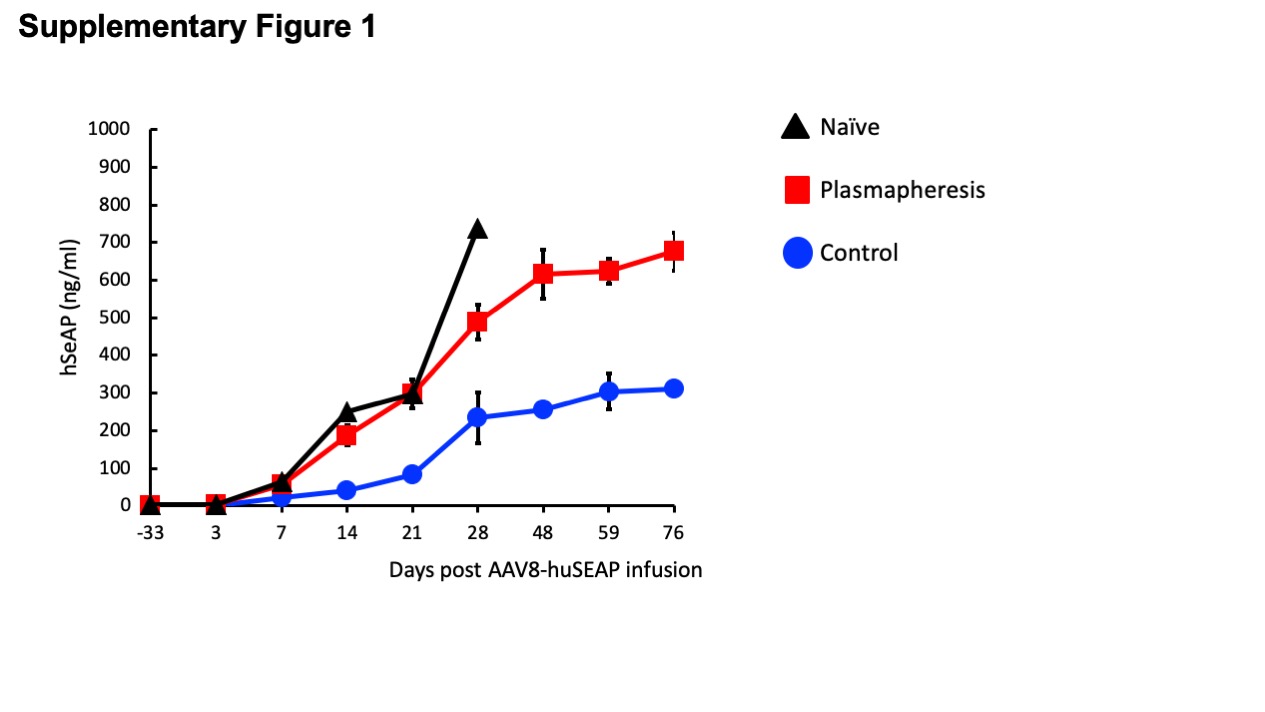
**

**Supplementary Figure 2**

**
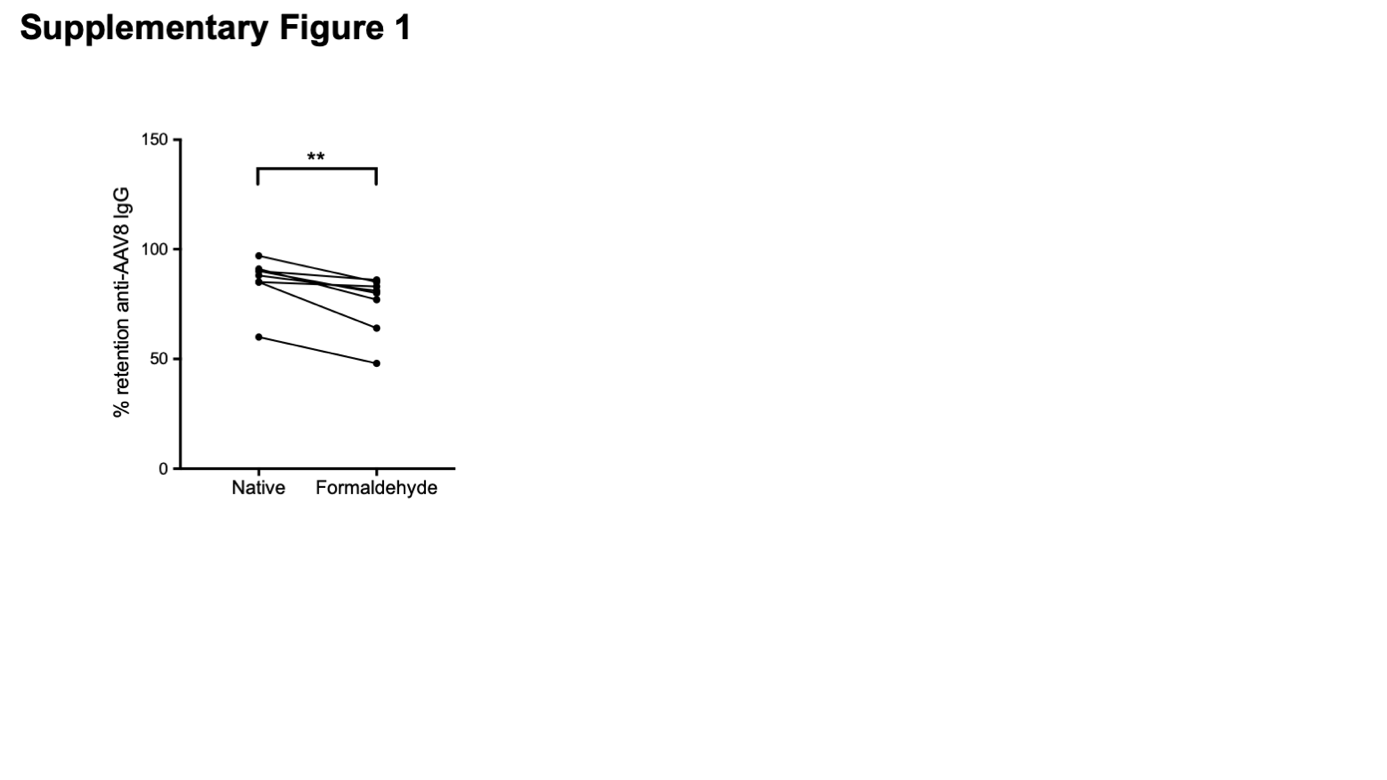
**

**Supplementary Figure 3**


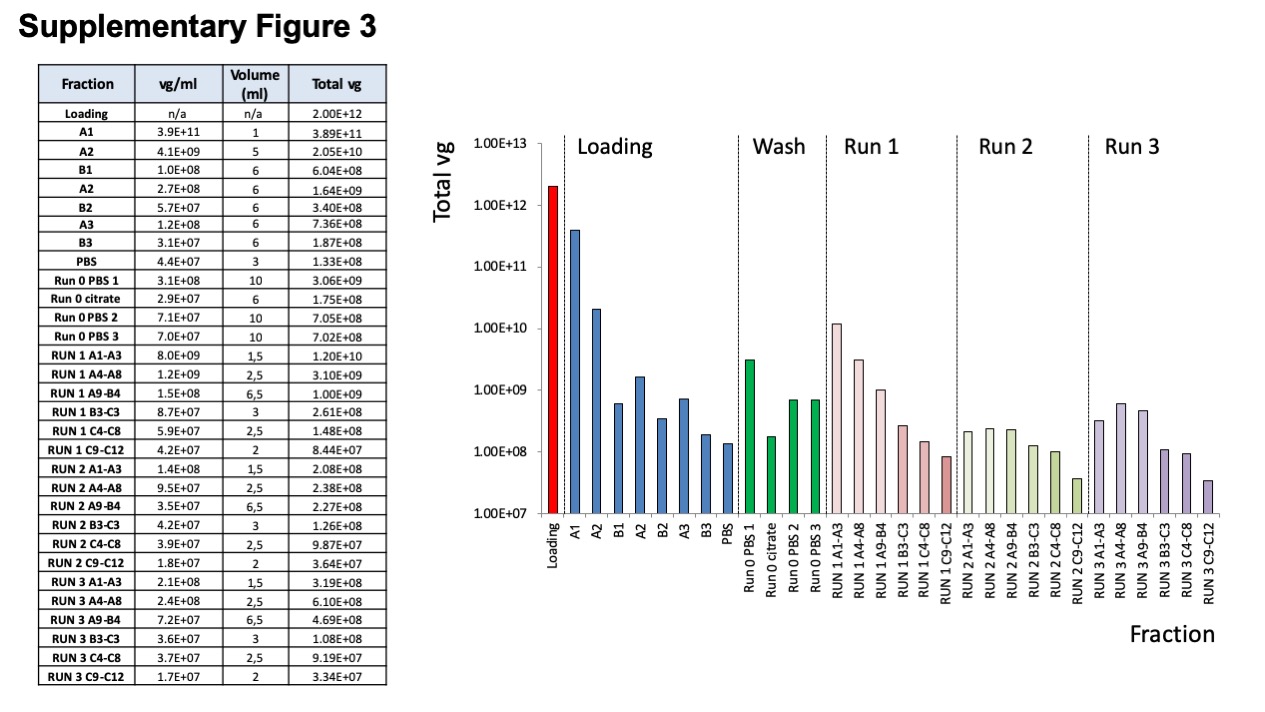

Supplement: Supplementary file 1 — Supplementary information. [file 41598_2020_57893_MOESM1_ESM.docx]
